# *Tylosema esculentum* (Marama Bean) as a Climate-Resilient Opportunity Crop: A Review of Nutritional, Medicinal, andGenomics Insights and Drought Adaptation

**DOI:** 10.3390/plants15203128

**Published:** 2026-10-13

**Authors:** Pfunzo Gavhi, Mulisa Nkuna, Gumani Luidney Nemauluma, Lindokuhle Mogwaduba, Nndamuleleni Romeo Murovhi, Takalani Mulaudzi

**Affiliations:** 1 Life Sciences Building, Department of Biotechnology, University of the Western Cape, Private Bag X17, Bellville 7535, South Africa; 4221352@myuwc.ac.za (P.G.); 4078438@myuwc.ac.za (M.N.); 4467547@myuwc.ac.za (G.L.N.); 4139811@myuwc.ac.za (L.M.); 2 Agricultural Research Council—Tropical and Subtropical Crops, P.O. Box 247, Levubu 0929, South Africa; romeo@arc.agric.za

**Keywords:** domestication, drought, food security, genetics, *Tylosema esculentum*, marama bean, climate-resilient, orphan crops, opportunity crops

## Abstract

*Tylosema esculentum* (Burch.) A. Schreib. (also known as marama) is a perennial legume native to the arid and semi-arid regions of southern Africa, where it thrives under extreme climatic conditions, particularly in hot and water-limited environments and nutrient-poor soils. This review outlines current knowledge on marama’s nutritional and medicinal potential, as well as genomic insights and drought adaptation mechanisms, to ensure sustainable food systems. Marama has added advantages since the entire crop, including grains/beans, stems and tubers, can be used as a source of food for animals and humans. However, the nutritional value and safe consumption of different tissues are dependent on developmental stages and appropriate processing to reduce anti-nutritional compounds and improve digestibility. Marama beans possess exceptional nutritional value, being rich in protein; unsaturated lipids; essential micronutrients; and health-promoting phytochemicals including phenolics, flavonoids, saponins and phytosterols. To date, the chloroplast and mitochondrial genomes of *Tylosema esculentum* (marama) have been sequenced and assembled, while its nuclear genome size has been estimated at approximately 1 gigabyte, providing valuable genomic resources for further research. Marama exhibits a predominantly drought-avoidance strategy characterized by the development of deep tubers for water and nutrient storage, reduction in transpiration through leaf senescence and stomatal regulation, and maintenance of plant water status during prolonged dry periods. Marama also exhibits osmotic adjustment, which further enhances cellular protection and stress tolerance under water-deficit conditions. Despite these promising attributes, marama remains largely underutilized due to limited agronomic data to support its cultivation and insufficient data on its metabolic and genetic architecture that drives drought adaptation and socio-economic potential. Thus, there is an urgent need for integrated research efforts that will facilitate the transition of this crop from a wild-harvested resource to a sustainable, climate-resilient crop suited to arid and semi-arid agroecosystems.

## 1. Introduction

Agriculture in arid and semi-arid regions is threatened by water scarcity, rising temperatures and erratic rainfall patterns, all of which intensify drought stress and constrain food production [1]. Neglected and underutilized crop species (NUCs), often referred to as orphan crops or opportunity crops, are increasingly recognized for their potential to complement existing staple crops and contribute to food and nutritional security in developing countries, particularly across Africa. Their neglect is evident in their classification as crops of low economic importance and in the lack of attention they receive from crop improvement and breeding programs [2,3].

Like landraces of major food crops, orphan legumes have genetically adapted to their natural environments over generations, largely in the absence of modern systematic breeding and selection [4]. The unique adaptative strategies exhibited by legumes provide a significant advantage under drought conditions through the evolution of a range of coordinated morphological, physiological, biochemical and molecular mechanisms that confer tolerance and enable survival in harsh and water-limited environments [1,5]. Therefore, a comprehensive understanding of these strategies, including key adaptive and stress-responsive traits, will provide valuable insights for plant breeders to develop crop varieties with enhanced drought tolerance, increased yield, improved seed quality and reduced dormancy [6].

Some widely cultivated legumes, such as the common bean (*Phaseolus vulgaris*), soybean (*Glycine max*), pigeon pea (*Cajanus cajan*) and faba bean (*Vicia faba*), generally perform poorly in severe water-limited environments and often require rainfall exceeding 400 mm per year to achieve reasonable yields [7]. These limitations have prompted a growing interest in orphan legumes, including grass pea (*Lathyrus sativus*), Bambara groundnut (*Vigna subterranea*), lima bean (*Phaseolus lunatus*), African yam bean (*Sphenostylis stenocarpa*), and marama bean (*Tylosema esculentum*). These legumes are valued for their natural adaptation to harsh environments and their ability to maintain growth and productivity under relatively low-input conditions, particularly in marginal and drought-prone environments [7,8]. Their nutritional potential also makes them promising candidates for the diversification of food sources and as contributors to more sustainable and climate-resilient food systems [9,10]. Among these legumes, marama bean stands out, owing to its extensive drought tolerance and high nutritional content [7].

**Figure 1:**
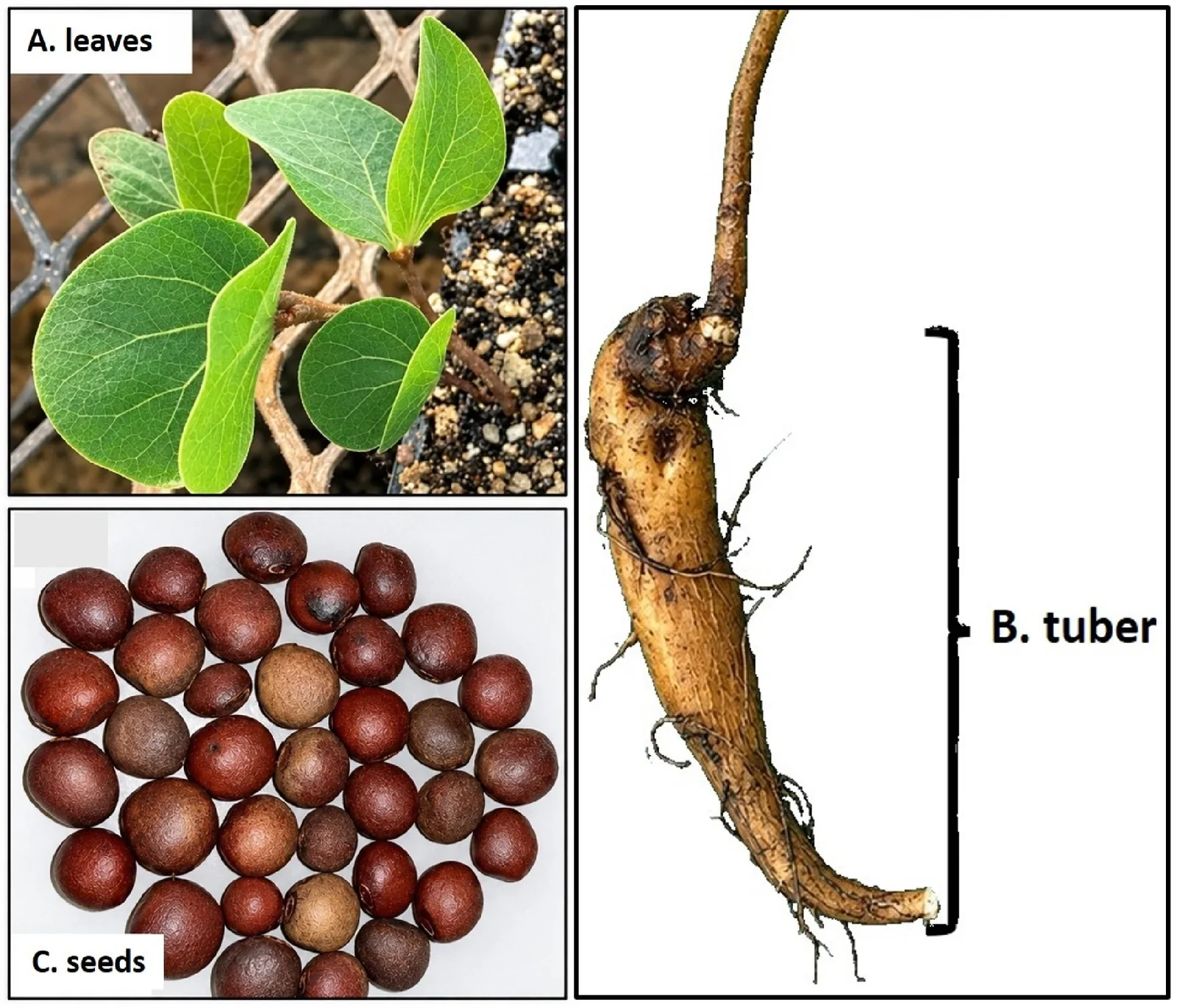
Leaves (**A**), tuber (**B**) and seeds (**C**) of *Tylosema esculentum* (marama bean). Pictures (own sources); seeds were bought from Livingseeds Heirloom Seeds (Pty) Ltd., Meyerton, Gauteng South Africa.

*Tylosema esculentum* (Burch.) (marama) A. Schreib. is a legume indigenous to the Kalahari Basin and neighbouring arid regions of Namibia, Botswana and South Africa [11]. The legume belongs to the Fabaceae family and the Cercidoideae subfamily [7]. Marama grows as a sprawling perennial vine (Figure 1A) in deep, sandy, and nutrient-poor soils where annual rainfall is below 400 mm and summer temperatures can exceed 37 °C. Marama is characterized by a large underground tuber (Figure 1B), weighing approximately 1 kg in young plants and exceeding 10 kg at maturity [7]. The tuber serves as an important storage organ and may facilitate survival and regeneration following prolonged drought or above-ground die-back [7]. This ecological profile reflects a highly specialised plant that can adapt to extreme environments. Marama has long been used by indigenous communities, with historical records as early as 1824 documenting the consumption of its seeds (Figure 1C) and tubers in the Kalahari Desert. Locally, the legume is known by various names, including marama bean, morama, braaiboonjie and camel’s foot in South Africa, Botswana and Namibia respectively [12]. Nutritionally, its seeds are highly nutrient-dense, containing approximately 30–40% protein and 35–48% lipids, with reported concentrations varying according to plant developmental stage and processing conditions. Moreover, multiple parts of the plant, including the seeds, stems and tubers, can be utilized as food [13]. The marama seed is enclosed in a hard, dark seed coat (Figure 1C) that enhances protection and longevity, but it imposes physical dormancy, thereby presenting challenges for germination and processing [14].

Owing to its unique features of high nutritional content and drought adaptation, marama is recognised as a source of food and a medicinal compound in southern Africa and is therefore referred to as the “green gold of Africa” [13,15].

Despite these exceptional qualities, marama remains largely undomesticated, with most plant materials predominantly harvested from wild populations rather than cultivated under formal agricultural systems. Transforming marama from a neglected wild plant into a viable cultivated crop requires a deeper understanding of its nutritional and medicinal potential, agronomic performance, genetic insights and drought response mechanisms. This review summarizes current knowledge on the utilization of the marama plant as a potential source of highly nutritious food and bioactive medicinal compounds. In addition, it expands on marama’s cultivation, genetic architecture and drought response mechanisms to inform breeding strategies for its potential domestication. Furthermore, the review identifies key knowledge gaps and research priorities required to facilitate the development and exploitation of this unique legume as a climate-resilient, nutrient-rich crop for sustainable agricultural systems in arid and semi-arid regions.

## 2. Literature Search and Selection

A comprehensive body of literature was retrieved from reputable academic databases, including Scopus, Web of Science, JSTOR, Google Scholar, Pubmed and AGRIS, for the review. The search strategy employed a combination of keywords and Boolean operators (OR, NOT and AND) to recover relevant published literature on “*Tylosema esculentum*”, “marama bean”, “marama”, “morama”, “nutrition”, “nutritional composition”, “cultivation”, “agronomy”, “growth”, “genetics”, “genomics”, “genetic diversity”, “transcriptomics”, “drought”, drought tolerance”, “drought stress”, “water stress”, “climate resilience”, and “adaptation”. The search was confined to journals and articles published in English to ensure the coverage of all relevant studies. Studies published in languages other than English and studies focusing on *Tylosema* species other than *T. esculentum* were considered outside of the scope of this review and were therefore excluded.

## 3. Nutritional Value of Marama

The nutritional composition of marama is well documented, with most studies confirming that the marama beans are highly nutritious [16]. Among indigenous legume crops, marama is highly versatile, with multiple plant tissues, including the seeds (grains), stems and tubers (Figure 1), being used as food. This versatility has contributed to its continued use in traditional food systems and its growing interest in modern food applications [13,16].

### 3.1. Marama Grains

Marama bean protein and oil content are comparable to those of conventional legumes such as soybean and groundnut and higher than those of several emerging underutilized legumes, as shown in Table 1. Dry-roasted, mature, edible seeds are a good source of protein (Table 1), containing essential amino acids such as histidine, leucine and lysine, among others [17]. Freshly harvested mature marama seeds are consumed after peeling and boiling; this is said to be the main traditional processing method across different regions [11]. Conversely, mature seeds are traditionally consumed by rural communities in southern Africa either boiled, roasted as a snack, or ground into flour for the preparation of porridge and beverages [11]. The flour has also been incorporated into baked products and dietary supplements [18,19].

The marama bean has a high lipid content (Table 1), characterized by health-beneficial fatty acids, particularly oleic, linoleic, palmitic and stearic acids [20]. Its high lipid content allows for the extraction of alternative plant-based unsaturated oil [18] that can be used for cooking and has the potential for commercial production, especially as a heart-healthy alternative to saturated fats. The bean contains a significant amount of carbohydrates and dietary fibre defatted flour, as shown in Table 1 [20]. Another food product processed from the marama bean is milk, an emerging product that, like soya milk, is currently being studied as a potential dairy alternative for lactose-intolerant populations [10,16]. The marama bean is also a valuable source of micronutrients, including calcium, iron, zinc, and magnesium (Table 1). In addition, the seeds contain a significant amount of vitamin B6, highlighting their potential contribution to dietary micronutrient intake [13,20]. Although marama bean is rich in micronutrients, the presence of antinutritional factors, such as trypsin inhibitor (245.45 TUI/mg DM), tannins, elastase inhibitor and cyanogenic glycoside, may limit nutrient bioavailability [28]. Alabi et al. [29] reported that the inclusion of raw, full-fat marama bean meal in broiler diets impaired growth and carcass characteristics because of the high levels of antinutritional factors, particularly trypsin inhibitors and phytic acid [29]. To maximize the utilization of the marama bean as a dietary protein source and to significantly reduce trypsin inhibitor activity in marama grain, cooking for longer than 30 min is recommended [28]. An ethnobotanical survey conducted by Gwamba et al. [11] in Botswana found that Batswana people boiled fresh marama bean for 10 to 30 min in water, while dry seeds were roasted in hot sand overnight or until golden brown. Importantly, marama beans exhibit a long shelf life, with traditional reports indicating a storage period of up to 60 months when properly stored in cool, dry environments using suitable containers, polypropylene bags, and jute sacks [11]. Further research and promotion of this legume are essential to harness its full potential in addressing global nutritional needs and ensuring food security.

### 3.2. Marama Leaves and Stems

To date, information on the nutritional composition and food uses of marama leaves and stems remains limited. Immature and mature marama stems are edible and have traditionally been consumed cooked as a vegetable or incorporated into soups in some parts of southern Africa, particularly in arid regions where few crops survive [12,13]. Although raw marama leaves are not commonly consumed, ethnobotanical reports indicate that they may be cooked or boiled prior to consumption, which improves their palatability by reducing their unpleasant odour and slimy texture [11]. Cooking may also contribute to the reduction of anti-nutritional compounds, although this aspect requires further experimental validation. In addition to its potential as a human food source, marama biomass is regarded as a good source of fodder for livestock [13].

### 3.3. Marama Tubers

The underground part of marama produces a large, nutrient-rich storage tuber that is noted for its substantial size compared with several conventional staple root and tuber crops, including potatoes, yams and sugar beets [30]. Young marama tubers, typically harvested at 1–2 years of age and weighing about 1 kg, are sweet, succulent and nutritious and can be consumed raw, boiled or roasted. As the tuber develops, it increases considerably in size, with a mature tuber (older than 2 years) potentially exceeding 10 kg in weight and containing up to 90% water. These tubers become highly fibrous and develop a bitter taste, limiting direct consumption; however, they may still be of value for livestock feed, starch recovery, bioenergy production and other industrial applications [13,15,30,31].

## 4. Medicinal Value of Marama

Marama has considerable potential for exploration in non-food applications, including its use as a source of medicinal compounds and in the cosmetic industry. Evidence supporting the medicinal potential of marama is predominantly sourced from ethnobotanical reports, in vitro studies and phytochemical investigations, which will be discussed in this section.

### 4.1. Traditional Medicinal Uses of Marama

Traditionally, extracts of marama beans and tubers have been used by communities in Southern Africa for several health benefits, including the treatment of diarrhoea and digestive disorders [15,32]. However, these traditional uses should be distinguished from experimentally proven therapeutic effects. Ethnobotanical have studies highlighted the use of marama leaf preparation in conjunction with other medicinal plant species such as *Bauhinia petersiana* and *Cucumis sativus* for the treatment of digestive disorders and other health-related purposes such as improving children’s appetite and increasing milk production in pregnant women [33]. Although there is limited knowledge regarding the medicinal use of marama tubers, Adeboye et al. [31] reported high dietary fibre, comparable to that of sweet potato, and suggested that marama tubers can serve as potential prebiotics [31]. A survey conducted in selected villages in Botswana reported that the traditional medicinal use of the marama bean is regarded as sacred and known only to a limited number of community members, such as traditional healers and traditional birth attendants [11]. Mature marama tubers are traditionally used by the Batswana people to cure diarrhoea, headaches and stomach pains and to manage hypertension, while pounded tubers and leaves are believed to improve women’s health in the postmenstrual cycle [33]. However, these traditional medicinal applications of marama tubers are based on ethnobotanical reports, and there is currently insufficient experimental and clinical evidence to establish their therapeutic efficacy, dosage, safety guidelines, and even their mode of action. Therefore, further research on the identification and characterization of active compounds and dose responses, as well as pharmacokinetic investigations; toxicology assessments; and, ultimately, well-controlled clinical studies on marama, is necessary.

### 4.2. In Vitro Antimicrobial and Cytotoxicity of Marama Bean Extracts

Increasing research interest in the potential bioactivity of *Tylosema esculentum* (marama) is emerging, particularly in the antimicrobial and cytotoxic properties of extracts from its different plant parts [34]. Chingwaru et al. [32] assessed antibacterial and anticandidal activity of *T. esculentum* against three methicillin-resistant bacterial strains (*Staphylococcus aureus*, *Mycobacterium terrae*, and *Corynebacterium diphtheriae*) and one fungal species (*Candida albicans*). Findings showed that ethanolic and methanol bean extracts exhibited higher potency against all tested microorganisms than aqueous extracts. Furthermore, the polyphenolic fractions exhibited high antimicrobial activity, as shown by low minimum inhibitory concentration (MIC) values, which ranged from 0.1 mg/mL to 1 mg/mL. These values are comparable to those of the reference antimicrobial agents used in this study, including amphotericin B (0.5 mg/mL) and cefsulodin (0.1 mg/mL) against *C. diphtheriae*, streptomycin (1.0 mg/mL) and gentamicin (0.5 mg/mL) against *M. terrae*, and amphotericin B (0.05 mg/mL) against *C. albicans* [32]. Seed oil extracts of marama exhibited selective antibacterial activity against *S. aureus*, with inhibition zones of 9.33 ± 0.58 mm, although this activity was significantly lower than that of positive controls tetracycline (36.67 ± 0.58 mm) and penicillin (27.33 ± 0.58 mm) [34]. Furthermore, there was no antibacterial activity observed against *Escherichia coli*, in contrast to positive controls tetracycline (25.00 ± 0.00 mm) and gentamicin (23.30 ± 0.58 mm) [34]. The cytotoxic effects of aqueous and ethanolic marama bean extracts, as well as aqueous tuber extracts, were evaluated using intestine epithelial cell lines of human (H4), porcine (CLAB), and bovine calf (CIBE) origin to asses protection against cytopathic rotavirus effects [35]. The extracts exhibited variable cytotoxicity depending on the plant part, extract type and cell line tested. Both aqueous and ethanolic marama bean and tuber extracts exhibited anti-rotavirus activity in vitro, with the seedcoat ethanolic extract showing the highest potency at concentrations of 0.001–0.01 mg/mL, particularly at 0.001 mg/mL [35]. Furthermore, cotyledon ethanolic extract also demonstrated significant activity at concentrations ≥0.1 mg/mL, whereas tuber aqueous extract was active at 0.1–0.01 mg/mL [35]. Overall, ethanolic extracts exhibited greater anti-rotavirus activity compared to the aqueous extracts [35]. This observed antiviral activity might be attributed to the release of nitric oxide from intestinal epithelial cells, suggesting that nitric oxide may contribute to the anti-viral effect, potentially through inhibition of viral nucleic acid replication and other mechanisms involved in viral infectivity [35]. These findings serve as preliminary evidence of the antiviral activity of marama and should not be interpreted as showing efficacy in the treatment of rotavirus infection in humans. Similarly, reported antimicrobial activity of marama extracts against bacterial and fungal pathogens should not be interpreted as therapeutic effectiveness.

However, there is insufficient scientific evidence using in vitro, in vivo, and clinical trials to support health claims associated with marama bean.

### 4.3. Phytochemical Composition of Marama

The potential health benefits of marama have been associated with the presence of diverse bioactive phytochemicals, including phenolic compounds and flavonoids [15].

#### 4.3.1. Leaves

Although the leaves of marama constitute an important above-ground component of the plant, their phytochemical composition remains poorly characterized. Most phytochemical investigations of marama have focused on the seeds, particularly the seed coat and cotyledon, as well as the tuber, with most phenolics and flavonoids identified as having potential antioxidant and other health-promoting activities. However, quanti-tative information on phenolic and flavonoid compounds and other secondary metabo-lites in marama leaves is currently limited, presenting a significant knowledge gap with respect to the potential contribution of leaf phytochemicals to the plant’s antioxidant and other biological activities.

#### 4.3.2. Seed/Grain

Lekalake et al. [36] reported total phenolic and flavonoid contents of 1.72 mg gallic acid equivalents (GAE)/g and 0.00023 mg catechin equivalents (CE)/g, respectively, in the marama bean [36]. In addition, Chingwaru et al. [15] reported several phenolic acids in acidified methanol extracts of marama bean cotyledons, including gallic acid (23.4 mg/100 g), protocatechuic acid (152.5 mg/100 g), syringic acid (12.7 mg/100 g), vanillic acid (20.5 mg/100 g), caffeic acid (38.9 mg/100 g), ferulic acid (8.9 mg/100 g) and sinapic acid (59.2 mg/100 g) [15]. These extracts also contained flavonoids, including catechin (11,5 mg/100 g), naringin (9.9 mg/100 g), hesperidin (919.1 mg/100 g), quercetin (23.5 mg/100 g), kaempferol (20 mg/100 g), rutin (25.7 mg/100 g) and fisetin (39.2 mg/100 g), which may contribute to the potential antioxidant and antimicrobial activities of marama [15]. Furthermore, another study reported different phenolic compounds identified in aqueous seedcoat extracts, including gallic acid (1.98 mg/g), 2,5-dihydroxyphenylacetic acid (0.49 mg/g), and p-Hydroxybenzoic acid (1.76 mg/g), while flavonoids, including Methyl(epi)catechin-3-O-gallate (5.84 mg/g) and Methyl(epi)afzelechin-3-O-gallate (8.34 mg/g), were also recorded [37]. Methyl(epi)catechin-3-O-gallate and methyl(epi)afzelechin-3-O-gallate have primarily been identified in green tea (*Camellia sinensis*) extracts, while their occurrence in legumes has not been widely reported [38]. However, despite the ethnobotanical and phytochemical evidence, experimental validation is limited.

#### 4.3.3. Tubers

Significant amounts of phytochemical compounds, particularly phenolics and flavonoids, have also been found in marama tubers. Kuvare et al. [39] reported total phenolic (4.48 ± 0.09 mg gallic acid equivalents (GAE)/g) and total flavonoid (0.93 ± 0.001 mg quercetin equivalents (QE)/g) contents in ethanolic extracts of marama tubers [39]. The tuber extract also demonstrated strong antioxidant activity, with a DPPH radical-scavenging IC_50_ value of 95.62 ± 7.08 µg/mL, showing its potential as a source of natural antioxidants [39]. Similarly, Chingwaru et al. [35] reported high concentrations of gallic acid and other phenolic substances in marama tubers, although quantitative concentrations of the individual compounds were not provided [35]. Another study also identified several bioactive compounds in marama, including phenolic compounds (gallic acid and protocatechuic acid), flavonoids (kaempferol and kaempferol-3-O-glucoside), griffonilide, and fatty acids (such as behenic acid) [40]. Although these findings show that phenolic and flavonoid compounds contribute to the phytochemical profile of marama tubers, quantitative information on individual phytochemicals remains limited, and comprehensive profiling of the tuber metabolome is still poorly understood. It is important to note that the reported health benefits of marama discussed in this review are largely derived from traditional knowledge and ethnobotanical studies. Evidence from controlled scientific studies remains limited, and therefore, these reported benefits should be regarded as traditional applications rather than established therapeutic effects. There is a clear lack of systematic investigations into its antimicrobial activity, as well as a limited number of in vivo studies using animal models to validate its reported immune-boosting effects. Although few studies have quantified non-enzymatic antioxidants such as flavonoids and phenolic compounds, no comprehensive metabolite profiling exists that integrates all plant tissues to fully characterise the distribution of antioxidants. This highlights knowledge gaps around marama bean studies that could support its importance in food, nutrition and medicinal product development.

## 5. Cultivation of Marama

Marama grows well in neutral (pH 7) soil with limited organic matter, low nutrients such as nitrogen and phosphorus and minimal soil water content [13]. Although marama’s optimum growth requires about 250–600 mm rain annually, it has been reported to grow in regions receiving a minimum of 100 mm of annual rainfall [13]. Marama is propagated from a seed, sown in soft, well-drained, weed-free soils [13]. Different seed treatment methods employed to break seed dormancy and enhance germination include mechanical scarification (involving seedcoat scratching using sandpaper) and chemical scarification using concentrated chemicals such as sulphuric acid [14]. Scarification also involves exposing the seed to low temperatures for prompt and uniform germination. Travlos et al. [14] investigated different seed pretreatment techniques to improve the germination of marama beans. These include soaking seeds in 95% concentrated sulphuric acid for 20 or 40 min; soaking in gibberellic acid (GA_3_) solution at 100 mg GA_3_ L^−1^ for 40 min, followed by thorough rinsing with running water; soaking in water at room temperature for 20 h; and mechanical scarification (untreated seeds with no chemical/mechanical pretreatment) [14]. While other pre-treatment methods result in intermediate final germination percentages, Bousquet [33] previously reported a germination rate of approximately 82% following scarification with industrial-strength sulphuric acid. Although mechanical scarification can disrupt the hard seed coat, thereby increasing seed permeability and facilitating water uptake and germination, excessive scarification may cause mechanical damage to the seed, compromising seed integrity and potentially increasing susceptibility to pathogen invasion [14].

Marama bean is preferably sowed at 5–20 cm depth into the soil, with one seed per hole [13]. Under favourable growing conditions, marama bean germination can take up to 21 days depending on the environmental conditions and seed pretreatment methods applied [13]. At the vegetative stage, marama grows as a vine-like plant, with its stem growing up to 3 m in length at full maturity [13]. The leaves are bilobed and soft in early growth stages but thicken and become greyish at full maturity [13]. Marama can be propagated vegetatively through its underground tubers, which serve as storage organs capable of re-sprouting and supporting plant regeneration [13,41,42]. Marama takes about 2–4 years to reach maturity in order to produce dependable seeds for future propagation [41,42]. However, based on these studies, the best cultivation practices for sustainable marama production are still limited; this is the main barrier hindering its sustainable domestication. Therefore, it is necessary to conduct further studies under controlled cultivation conditions to develop marama cultivation guidelines for its sustainable production.

## 6. Marama Bean Genetics

Genomic evaluation of marama is crucial for domestication and breeding strategies aimed at improving this indigenous leguminous crop. To date, both marama’s chloroplast and mitochondrial genomes have been successfully sequenced and assembled, with its overall nuclear genome size estimated to be approximately 1 gigabase [43,44].

### 6.1. Marama Chloroplast Genome

Kim and Cullis [43] reported a complete assembled chloroplast genome of approximately 161,537 bp in length, which contains a specific unique inversion of 7479 bp, including six genes that have not been identified in any other legumes—namely, *ribulose-1.5-bisphosphate carboxylase/oxygenase* (*rbcl*), *acetyl-coA carboxylase beta subunit* (*accD*), *photosystem I subunit I* (*psal*), *hypothetical chloroplast reading frame 4* (*ycf4*), *chloroplast envelope membrane protein A* (*cemA*), and *cytochrome f* (*petA*) [43]. As described with reference to other crops, some of these genes contribute to photosynthesis, carbon fixation, lipid metabolism, chloroplast functioning and plant development [45,46,47,48]. *rbcl* encodes the large subunit of rubisco (RuBisCO), the enzyme that catalyses CO_2_ fixation [45]; *accD* encodes the β/carboxyl-transferase (ACCase) complex and is reported to catalyse acetyl-CoA carboxylation and produce malonyl-CoA, which are necessary for fatty acid biosynthesis in the chloroplast [49]. *Psal* contributes to the assembly, stability and structural integrity of photosystem I and can influence plant adaptation to light stress [46]. *Ycf4* encodes proteins required for photosystem I assembly [47,48], whereas both *cemA* and *cytochrome f* (*petA*) play roles that are related to transport during homeostasis and energy generation in plants [43].

### 6.2. Marama Mitochondria Genome

Conversely, Li and Cullis [44] revealed that the size of the *Tylosema esculentum* (marama) mitochondrial genome is approximately 399,572 bp in length, which is larger than that of *Arabidopsis thaliana* (366,924 bp) although slightly smaller than the mitogenomes of several other fabaceae species, including *Vigna radiate* (402,558 bp), *Glycine max* (401,262 bp), and *Millettia pinnata* (425,718 bp) [44]. The GC content of the marama mitochondrial genome is 44.71%, which is comparable to that of *Arabidopsis thaliana* (44.8%), *Millettia pinnata* (45.4%), and *Vigna radiata* (45.1%).

Using MITOFY and AGORA organelle genome annotation platforms, 35 protein-coding genes, 3 rRNA genes, and 15 tRNA genes were identified from the assembled mitochondrial genome. Two protein-coding small-subunit ribosomal protein genes (*rps7* and *rps19*) were identified as pseudo genes, whereas other genes occur in duplicate, including ATP synthase genes (*atp1* and *atp8*) and the NADH dehydrogenase gene (*nad6*). Four protein-coding genes—namely, large-subunit ribosomal protein genes (*rpl2*, *rps2*, *rps11*, and *rps13*)—were absent from the marama mitochondrial genome.

Interestingly, several functional genes that have been reported to be lost during angiosperm evolution are retained in the marama mitochondrial genome. For example, *rpl10*, which is absent from the mitochondrial genomes of some Fabaceae species, including *Vigna radiata* and *Millettia pinnata*, is present in marama. Similarly, cytochrome c oxidase subunit *2* (*cox2*) and *rpl10* genes are reported to be absent from *Vigna radiate* and *Lotus japonicus* but are retained in marama. In addition, *sdh4* (*succinate dehydrogenase subunit 4*), which exists as a pseudo gene in *Glycine max* and *Millettia pinnata*, appears to be functional in marama. The retention of these functional genes may have important implications for mitochondrial and cellular functions. For instance, *cox2* plays an essential role in mitochondrial transport and energy metabolism, while *pl10* contributes to mitochondrial protein synthesis and is therefore important for plant growth, metabolism and adaptation to environmental stress [50].

### 6.3. Marama Nuclear Genome

Although early genomic studies on marama primarily focused on its chloroplast and mitochondrial genomes, recent advances have substantially improved our understanding of its nuclear genome. Li and Cullis [51] generated a high-quality nuclear genome assembly using 21.5 Gb of PacBio HiFi sequencing data using Sequel II platform (Pacific Biosciences, Menlo Park, CA, USA) combined with Omni-C scaffolding 9 [51]. This resulting assembly was 558.78-Mb, with a scaffold N50 of 22.68 Mb and a BUSCO completeness of 99.1% [51]. K-mer analysis supported a tetraploid genome organisation (2n = 4x = 44), with GenomeScope analysis estimating a genome size of approximately 277.44 Mb and revealing substantial heterozygosity [51]. Furthermore, this assembly contained 49343 predicted protein-coding genes, with transcriptomic support from the root and leaf tissues. The analysis further supported the interpretation of marama as an ancient allotetraploid, with k-mer coverage patterns indicative of its complex polyploid genome structure [51].

In parallel with genome assembly, molecular marker resources have been developed to facilitate the characterisation of genetic diversity in marama. Species-specific microsatellite markers were developed from genomic libraries [52] and subsequently used to assess the genetic variation within and among Namibian populations [53,54]. These studies revealed substantial microsatellite polymorphisms and genetic variation among natural populations, highlighting the underlying genetic diversity of the wild marama germplasm. More recently, whole-genome sequencing of 31 individual marama generated 23,772 high-quality biallelic SNPs and revealed two major genetic clusters. However, the phenotypic significance of this population structure remains poorly understood, particularly in relation to traits associated with drought adaptation, domestication, and agronomic performance [51]. Collectively, the available genomic and molecular marker resources provide an important foundation for the advancement of the genetic diversity and breeding of marama. However, several important gaps remain. These include the need for further improved haplotype-resolved genome assemblies, broader and geographically representative population sampling, genome-wide association and trait-mapping studies, and functional validations of candidate genes and variants. Addressing these gaps will be critical for linking genomic variation with key domestication, drought adaptation and agronomic traits and for accelerating the development of marama as a climate-resilient crop.

## 7. Drought Stress Adaptation Mechanisms in Marama

Drought is one of the major abiotic stresses affecting crop productivity, with severe water deficits capable of causing yield reductions of over 50% in some crops [55]. However, plants activate various morphological, physiological, biochemical, and molecular response mechanisms to ameliorate the effects of drought stress [56]. These mechanisms will be described to establish and propose the main traits responsible for drought tolerance in marama.

### 7.1. Morphological and Physiological Drought Responses

Based on currently available data, marama primarily employs a combination of morphological and physiological response traits to adapt to drought stress [7]. This is mainly due to the limited availability of biochemical and molecular drought-based studies. When growing under well-watered conditions, marama is characterized by numerous branches with runners extending along the ground. This vigorous vegetative growth supports the development of a large number of leaves and substantial biomass accumulation. Generally, under limited water conditions, roots experience reduced water uptake, leading to a decline in leaf water potential (LWP), increased abscisic acid (ABA) production, and subsequent stomatal closure. This response is beneficial, as it reduces transpiration, stabilizes shoot water status, and limits CO_2_ uptake and photosynthesis. Karamanos et al. [57] reported that marama exhibited the lowest stomatal conductance under dry conditions in both greenhouse and field experimental trials, suggesting a gradual stomatal closure that commenced at 11 h and reached maximum closure around 19 h, reflecting an adaptive drought response [57]. Under progressively drying soil, marama exhibited early stomatal closure, which occurred before substantial changes in leaf water potential, thereby reducing transpiration and contributing to the maintenance of leaf hydration [58]. Marama’s drought response is characterised primarily by conservative water-use strategies rather than enhanced photosynthetic water-use efficiency [58]. During dry periods, above-ground growth slows, as observed by a reduction in the leaf area and stem elongation [58]. As a strategy to minimize transpiration and water conservation, marama further exhibits the shedding of older leaves and senescence, reducing the transpiring leaf area while enabling a limited number of young leaves to remain functional [57]. These traits represent a drought-escape mechanism whereby marama prioritises lifecycle completion before drought conditions become unbearable [59].

High LWP under an extremely limited water supply was also reported in marama, suggesting that marama, as a tuberous legume, is able to maintain a stable LWP by drawing tuber-stored water [57]. A similar strategy has also been reported in other tuberous crops, such as cassava (*Manihot esculenta*), sweet potato (*Ipomoea batatas*), potato (*Solanum tuberosum*), and yams (*Dioscorea*), which have shown better adaptation to early-phase drought [60,61]. Furthermore, since LWP reflects the overall plant water status, maintaining a high LWP is associated with drought avoidance, an adaptation strategy in which plants maintain favourable water status under limited water availability through structural and physiological mechanisms [56]. Marama bean has been reported to develop a large underground storage organ (tuber) that serves as a reservoir for water and nutrients. The tubers can contain up to 90% water by fresh weight, enabling the plant to survive prolonged periods of drought [7,15]. These tubers remain viable during drought conditions and allow for rapid vegetative re-growth when conditions are more favourable [41].

The tuberous root also facilitates the uptake of moisture from deeper soil layers, thereby enabling the plant to survive extended dry periods [7]. Furthermore, marama also possesses an extensive tap root, which allows for penetration into deeper soil surfaces to access subsoil moisture [30]. This explains why marama can grow in sandy dry areas [61]. This is consistent with the study by Karamanos et al. [57], which reported that marama plants grown from tubers exhibited high relative leaf water content (LWC) under dry conditions compared with those grown from seeds [57]. In other tuberous crops such as potato, a deep and extensive root system contributes to drought response and tolerance by effectively facilitating root-to-shoot ratio transport of ABA, which induces stomatal closure [62]. Similarly, the roles of tubers as water storage organs and underground storage-based strategies for drought survival have been reported in other crops, including root and tuber crops [62,63,64]. This suggests that marama may possess multiple drought-adaptive traits, although evidence demonstrating a direct link between the ability to maintain high relative leaf water content (RLWC) and tuber size in this crop remains limited.

Although there is a paucity of detailed physiological studies examining parameters such as LWP, osmotic adjustment, and xylem vulnerability, available data suggest that marama conserves water loss aggressively and relies on tuber-stored water reserves rather than maintaining stomatal conductance under drought conditions.

### 7.2. Biochemical Drought Responses

Water deficit triggers a complex network of biochemical responses that are crucial for drought adaptation in plants. These responses help maintain cellular homeostasis, protect macromolecules from oxidative damage and sustain metabolic processes under limited water availability. In plants, biochemical responses include modulation of phytohormone signalling (as discussed Section 7.3), osmolyte accumulation, activation of antioxidant defense systems and alterations in stress-related metabolites. Collectively, these mechanisms enhance osmotic adjustment, preserve membrane integrity, and improve the plant’s ability to withstand prolonged periods of drought [65,66,67].

#### 7.2.1. Osmotic Adjustment

Osmotic adjustment is a well-studied phenomenon that is crucial for the plant’s drought tolerance. It is achieved through the accumulation of osmolytes such as proline, soluble sugars, glycine betaine, and organic acids in response to water deficit. The synthesis of compatible solutes contributes to drought adaptation by protecting cellular structures and metabolic processes, maintaining leaf turgor and preserving essential physiological functions, thereby enhancing plant tolerance to limited water availability [68,69]. Osmolytes further induce carbohydrate partitioning during the plant’s reproductive phase and improve average yield [70,71,72]. Metabolite profiles have previously been explored to gain insights into osmotic adjustment based on water relation parameters such as the solute potential at zero turgor; the relative water content at zero turgor; and the slope of the line relating pressure potential to relative water content, as demonstrated in the faba bean [70]. Similarly, Karamanos et al. [57] studied drought tolerance mechanisms using the same parameters on field-grown marama plants that were propagated from both seed and tubers and those grown in a greenhouse, propagated from seeds. The results demonstrated that marama exhibited a progressive osmotic adjustment under drought stress, reflected primarily in the progressive decline in solute potential at zero turgor (Ψs,o), suggesting enhanced solute accumulation [57]. This response was accompanied by changes in LWP, relative water content (RWC), stomatal conductance, leaflet angle, soil water content and water potential index, showing the coordinated regulation of plant water status and water loss [57]. Plants grown from tubers exhibited greater osmotic adjustment, as shown by more negative Ψs,o values, showing the ability to maintain plant water status under drought conditions [57].

In addition to its role in osmotic balance, proline acts as a scavenger of reactive oxygen species (ROS) [56], functions as a molecular chaperone (by protecting the plant’s protein integrity) and improves enzyme activity [71]. Under drought stress, tuberous crops accumulate proline as one of the primary osmolytes. Several crops, including potato and sweet potato, have demonstrated that drought-induced proline accumulation is positively associated with enhanced stress tolerance and may serve as a useful biochemical marker for the screening of drought-tolerant genotypes [73,74,75,76]. Despite a lack of studies showing a detailed link between osmolyte accumulation and osmotic adjustment in marama, preliminary data suggest the possible accumulation of osmolytes during desiccation based on a decline in solute potential at zero turgor [7,58]. Although phenolics have been identified in marama leaves and seed coat, as described in Section 4, the occurrence of these metabolites in marama tubers and their potential roles under drought have not been reported. Therefore, further research is needed to identify and characterize osmolytes, particularly proline, as well as other metabolites in marama tubers and to elucidate their roles in maintaining tuber function and plant survival under water stress.

#### 7.2.2. Antioxidant Defense System

Plants also activate antioxidant defense systems comprising both enzymatic and non-enzymatic antioxidants. Enzymatic antioxidants include superoxide dismutase (SOD), ascorbate peroxidase (APX), catalase (CAT), peroxidase (POD), glutathione reductase (GR), and enzymes of the ascorbate–glutathione cycle, whereas examples of non-enzymatic antioxidants include ascorbic acid (AsA), glutathione (GSH) and glutathione disulfide (GSSG). Together, these antioxidant components play crucial roles in mitigating oxidative damage and enhancing drought stress tolerance by scavenging ROS [56]. To date, apart from the report that suggested the accumulation of antioxidants during desiccation in marama [7], the role of the antioxidant defence system in drought tolerance remains largely unexplored. In particular, no studies have simultaneously characterised the involvement of antioxidant enzyme activity and the expression of genes encoding these enzymes in marama under drought tolerance. Thus, it remains unclear whether drought-induced oxidative stress in marama is associated with the activation of enzymatic antioxidant signalling. However, studies in other drought-tolerant tuberous (sweet potato) [72], and legume (soybean, cowpea, and African yam) crops reported increased activities of SOD and APX under drought stress [75]. However, it has yet to be established whether similar biochemical and molecular responses occur in marama. Addressing this knowledge gap through integrated biochemical and transcriptomic analysis would assist in determining the possible contribution of antioxidant defence to drought tolerance in marama and identify candidate antioxidant enzymes and genes that may be responsible for its ability to withstand drought.

### 7.3. Molecular Drought Responses

Drought-induced changes in gene expression often overlap with hormone-regulated signalling pathways. Several studies suggest that reduction in a plant’s cell turgor is one of the main physiological responses to water deficit, triggering a cascade of molecular adaptations that regulate gene expression and enhance drought tolerance [76]. Abscisic acid (ABA) is the primary phytohormone inducing short-term responses such as stomatal closure and long-term responses that promote root growth and enhance water uptake [77,78]. Other studies have demonstrated that stomatal conductance is also dependent on other endogenous phytohormones, such as auxin, cytokinin, ethylene and salicylic acid, among others [79,80,81]. Recent transcriptomic evidence provided insight into the molecular response of *Tylosema esculentum* to drought [82], with the study identifying 144 differentially expressed genes (DEGs) on drought-stressed marama plants, of which 91 were upregulated and 53 were downregulated. These genes were associated with pathways involved in water-deficit responses, chloroplast organization, transcriptional regulation, ABA signalling and mitogen-activated protein kinase (MAPK) signalling. Furthermore, RT-qPCR confirmed the overexpression of *phospholipase C* (*PLC*), *purple acid phosphatase* (*PAP*), *PAP17*, *phosphatase 2C* (*PP2C*), *PP2C77* and *aldehyde dehydrogenase* (*ALDH*) [82]. Among these genes, *PLC* exhibited the greatest expression and may contribute to drought signalling through phospholipid-mediated Ca^2+^ signalling. Similarly, *PAP* and *PAP17* may contribute to phosphate remobilization and oxidative stress [82]. Furthermore, the upregulation of *PP2C* isoforms is consistent with the established role of ABA-dependent stress signalling, whereas upregulation of *ALDH* may facilitate the detoxification of reactive aldehydes generated during lipid peroxidation [82].

In addition to transcriptomic responses, physiological and biochemical traits of marama may also contribute to its adaptation to water-deficit stress. For example, marama Rubisco exhibited a lower apparent K’m for CO_2_ than wheat, suggesting a potential adaptation of carbon fixation to high-temperature conditions [58]. However, the predicted photosynthetic advantage was relatively small, indicating that morphological and stomatal mechanisms may play more crucial roles in drought survival in marama [58]. Furthermore, genes identified in marama organellar genomes, including *petA* in the chloroplast genome and *rpl10* in mitochondrial genome, may have a biological relevance to stress response [82]. This potential relevance is supported by the established roles of *petA* in photosynthetic electron transport and *rpl10* in ribosomal function. A study by Defez et al. [83] showed that *Medicago sativa* plants overexpressing *auxin indole-3-acetic acid* (*IAA*) accumulated 25% more Rubisco large subunit (RbcL) protein under drought stress than wild-type plants. The study also demonstrated significant upregulation of thylakoid protein PetC and relatively minor changes in PetA content in *M. sativa* under drought stress. These proteins are associated with dissipating light energy and reduced efficiency of photochemical reactions in photosynthesis, which, in turn, protect plants from drought-induced severe damage [83].

Another candidate gene identified in the marama mitochondrial genome, *RPL10*, has also been implicated in stress responses in other plant species. In tobacco (*Nicotiana benthamiana*), *RPL10* was reported to contribute to drought tolerance via the ABA signalling pathway [84], while in rice (*Oryza sativa*), it has been associated with maintenance of the 80S ribosomal functions and the regulation of plant cellular translational activities [85]. However, unlike drought-responsive genes *PLC*, *PAP*/*PAP17*, *PP2C*, and *ALDH* identified in marama, the transcriptional responsive and functional contributions of *RPL10* and other organellar candidate genes to drought tolerance in marama have not yet been demonstrated. Their presence in marama organellar genomes should therefore be regarded as a potential basis for future investigation rather than direct evidence of drought adaptations.

Integrating these organellar candidate genes with the recently identified drought-responsive nuclear genes provides an opportunity to investigate the molecular basis of drought adaptation in marama using transcriptomic, genomic and functional approaches. In particular, future studies should determine whether these organellar genes are differentially expressed or otherwise functionally regulated under water deficit and whether their activities contribute to the physiological and molecular mechanisms underlying drought tolerance in marama.

## 8. Summary of Drought Adaptation Mechanisms in Marama

**Figure 2:**
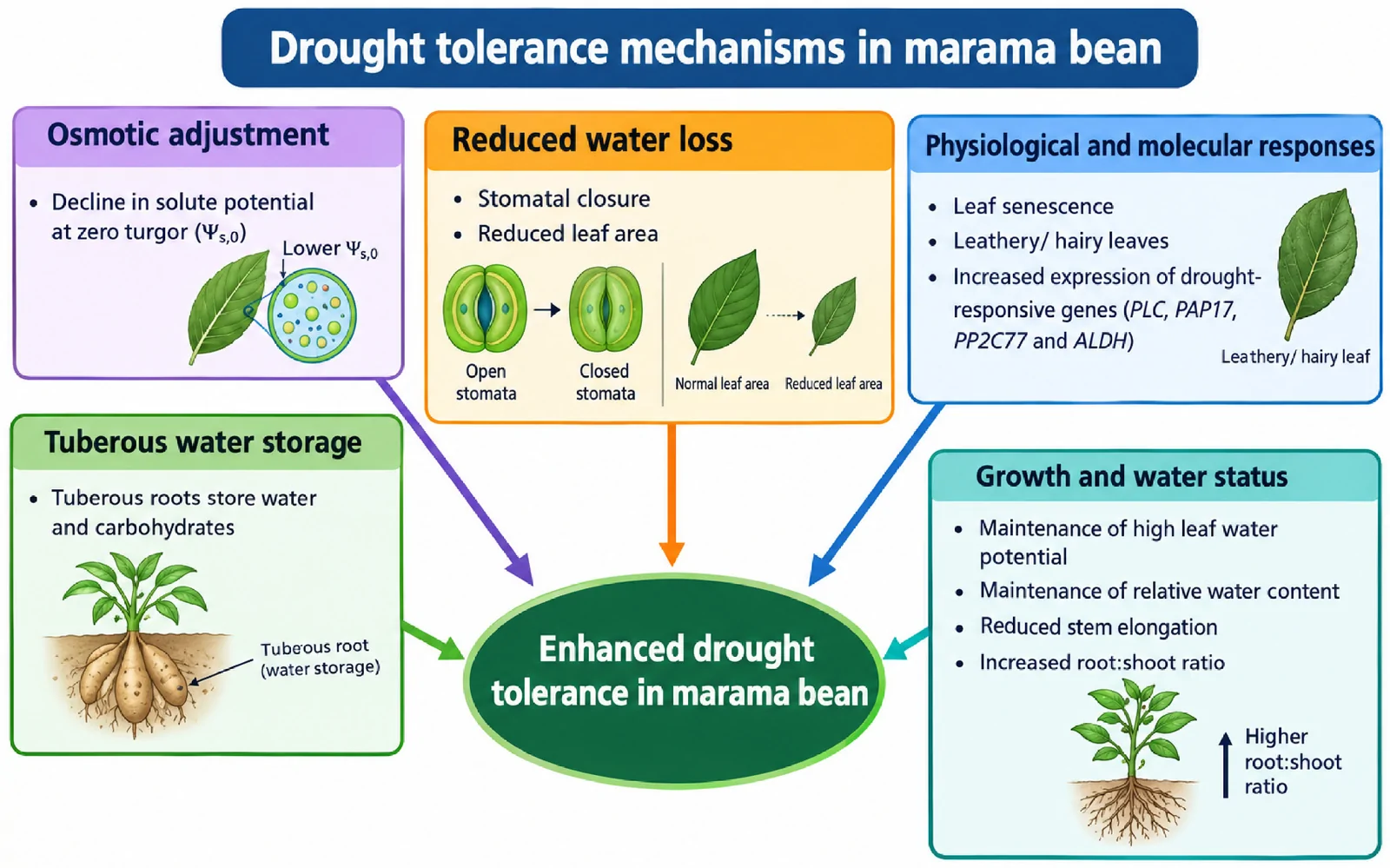
Summary of marama’s potential drought tolerance mechanisms. These mechanisms include reduced water loss, growth traits and improved water status through maintained leaf water potential and tuberous water storage, increased expression of drought responsive genes and osmotic adjustment.

*Tylosema esculentum* (marama bean) exhibits unique drought adaptation strategies involving morphological, physiological, biochemical, and putative molecular responses that collectively contribute to drought avoidance, escape and tolerance (Table 2, Figure 2). Morphologically, marama minimizes transpiration to prevent excess water loss through the reduction in leaf area; leaf shedding and senescence; the thickening of older leaves, forming a leathery texture (escape); reduced stem elongation; and increased root:shoot ratio/tap root elongation (drought avoidance). Marama’s main physiological strategy involves maximal storage of water and nutrients in the tubers and later drawing from the tuber under drought conditions. In addition, marama minimizes water loss through reduced stomatal conductance (stomatal closure) and maintenance of LWP and RWC (drought avoidance). Biochemically, marama maintains a progressive osmotic adjustment. Furthermore, emerging transcriptomic evidence suggests that marama responds to drought stress through the coordinated regulation of stress signalling, ABA-related, phosphate-metabolism and oxidative stress pathways, with *PLC*, *PAP*, *PAP17*, *PP2C77* and *ALDH* emerging as potential drought-responsive candidate genes.

## 9. Conclusions and Future Directions

As climate change is predicted to intensify the effects of drought, wider utilisation of opportunity crops is key. Marama offers a promising opportunity for crop diversification and resilience in marginal agroecosystems. This is due to its exceptional nutritional profile and potentially unique combination of traits that enable the crop to thrive and produce in arid, sandy soils. However, this review identified several gaps in understanding drought adaptation strategies in marama, including the lack of comprehensive studies on validated agronomic trials, food safety, limited nuclear genomic resources, reproductive biology, germplasm conservation, socio-economic assessment and standardized drought experiments focusing on transcriptomics and metabolomics. In addition, drought-related studies must evaluate drought-induced survival strategies under both controlled greenhouse and open field conditions to develop cultivation guidelines for diverse agroecological environments and assess the crop’s socio-economic value to support adoption by farmers and other stakeholders. Thus, future research must include coordinated, multidisciplinary efforts, including the integration of omics investigations, to uncover molecular mechanisms linking metabolites, gene expression, and drought-adaptive phenotypes.

## Figures and Tables

**Table 1:** Comparative nutritional composition of marama bean and selected legume crops.

Nutrient Content	Selected Legumes							
	Roasted Marama Bean [20]	Raw Bambara Groundnut [21]	Roasted Lima Bean [22]	Dry African Yam Bean [23]	Roasted Sword Bean [24]	Raw Fava Bean [25]	Dry Cowpea [26]	Dry Green Gram [27]
Proximate (g/100 g)								
Moisture	-	-	-	9.25	-	-	11.95	9.75
Protein	34.71	19.20	19.79	22.46	28.33	25	23.52	27.50
Fibre	3.94	20.92	5.02	158	7.36	1.672	10.60	4.63
Fats	40.06	6.34	1.05	4.35	9.85	1.2	1.26	1.85
Carbohydrates	11.85	61.30	62.62	53.68	44.06		60.3	62.3
Minerals (mg/100 g)								
Ca	241	0.04	216.14	15.00	3.60	104	110	112.60
P	454	0.29	302.10	99.00	2.70	357	424	415
Mg	274.5	0.17	306.21	69.00	1.60	4.2	184	150.20
K	895	1.40	955.12	419.00	5.10	9.76	1112	1725
Zn	6.2	0.23	4.10	0.78	57.32	3.1	3.37	3.28
Fe	3.95	0.33	7.68	1.50	4.06	1.8	8.27	5.05
Vitamins (mg/100 g)								
B2	0.06	0.31	0.20	0.20	1.8	-	-	0.32
B12	0.004	-	-	-	-	-	-	-
B6	1.71	0.44	0.47	0.10	-	-	-	1.18
Thiamine (B1)	0.38	0.61	0.502	0.19	0.8	-	-	0.88

Marama bean (*Tylosema esculentum*); Bambara groundnut (*Vigna subterranea*); Lima bean (*Phaseolus lunatus*); African yam bean (*Sphenostylis stenocarpa* Hochst ex. A. Rich); Sword bean (*Canavalia gladiata*); Faba bean (*Vicia faba* L.); Cowpea (*Vigna unguiculata* L. Walp); Green gram (*Vigna radiata*). “-” means data not available.

**Table 2:** Drought response traits in *Tylosema esculentum.*

Plant Material	Origin	Trial	Plant Age	Water Treatment	Main Findings	Limitation	Ref
**Leaves**	Greece	Open field	130 days	Drip irrigation by 1.2 mm at 10- and 5-day intervals under rainout shelters	Low leaf water potential, soluble potential at zero turgor, reduced RWC and low stomatal conductance	Lack of integration of morphological, physiological, biochemical and molecular analysis for robust drought tolerance screening	[58]
**Leaves**		Greenhouse	Sampled at different ages, from 26 to 105 days after sowing	Different water levels (50 mL; 100 mL; 200 mL and 400 mL irrigation water per pot)			
**Leaves**	ND	Greenhouse	5 months and 20 days	10–50 days of water withholding	Progressive soil drying induced stomatal closure and ceased photosynthesis	The study only investigated one drought level and did not clearly show the time	[59]
**Leaves, stem and tubers**	Greece	Greenhouse	Sampled at different ages, from 26 to 105 days after sowing	Different water levels (50 mL; 100 mL; 200 mL and 400 mL irrigation water per pot)	Drought stress restricted vegetative growth and total dry matter production	Greenhouse trial with no field trial validation, limited biochemical and physiological characterization and study focused only on vegetative growth rather than reproductive development and yield	[41]
**Leaves**	Namibia	Greenhouse	3 months and 22 days	4 weeks of water withholding	Reduced shoot length, number of open bilobed leaves and RWC; upregulation of drought-responsive genes, chloroplast organisation and transcriptional regulation genes	Limited biochemical and physiological characterization and lack of field trials to validate the greenhouse findings	[84]

Key: ND (not determined); RWC (Relative water content).

## Data Availability

The original contributions presented in this study are included in the article material. Further inquiries can be directed to the corresponding author.
